# Influence of right ventricular pacing on mitochondrial function in adults

**DOI:** 10.14814/phy2.70568

**Published:** 2025-10-02

**Authors:** Pawut Gumrai, Teerapat Nantsupawat, Nattayaporn Apaijai, Arintaya Phrommintikul, Narawudt Prasertwitayakij, Siriporn C. Chattipakorn, Nipon Chattipakorn, Wanwarang Wongcharoen

**Affiliations:** ^1^ Division of Cardiology, Department of Internal Medicine, Faculty of Medicine Chiang Mai University Chiang Mai Thailand; ^2^ Cardiac Electrophysiology Research and Training Center, Faculty of Medicine Chiang Mai University Chiang Mai Thailand; ^3^ Cardiac Electrophysiology Unit, Department of Physiology, Faculty of Medicine Chiang Mai University Chiang Mai Thailand; ^4^ Center of Excellence in Cardiac Electrophysiology Chiang Mai University Chiang Mai Thailand; ^5^ The Academy of Science The Royal Society of Thailand Bangkok Thailand

**Keywords:** mitochondrial function, oxidative stress, permanent pacemaker, right ventricular pacing

## Abstract

Right ventricular pacing (RVP) frequency and duration significantly associate with RVP‐induced cardiomyopathy. However, human data regarding RVP burden and mitochondrial function is scarce. We aimed to investigate the association between the duration and percentage of RVP and mitochondrial function in the peripheral blood mononuclear cells (PBMCs). This cross‐sectional study enrolled individuals with a permanent pacemaker device with RVP capabilities, implanted for over 1 month, and exhibiting preserved left ventricular (LV) systolic function. The assessment of mitochondrial function was conducted using PBMCs to explore its correlation with RVP burden since pacemaker implantation. The analysis included 96 patients. The majority of patients had advanced or third‐degree atrioventricular block, with a median RVP percentage of 84% and a median duration since implantation was 64 months. Substantial RVP percentage (RVP ≥ 40%) was independently associated with increased cellular oxidative stress (*p* = 0.015). Patients with longer RVP exposure (duration since implantation ≥ 60 months) were independently associated with increased mitochondrial oxidative stress (*p* = 0.030). Both associations remained significant after adjusting for confounding variables. In individuals with permanent pacemaker and preserved LV systolic function, substantial RVP percentage and prolonged RVP exposure were independently associated with increased cellular and mitochondrial oxidative stress.

## 1 INTRODUCTION

Permanent cardiac pacemaker (PPM) is a standard therapy for the management of bradycardia, particularly in individuals with sinus node dysfunction (SND) or atrioventricular block (AVB) (Glikson et al., 2022; Kusumoto et al., 2019). The conventional location for ventricular pacing is the right ventricle, commonly at the apical or septal area (Bongiorni et al., 2013). As a consequence of non‐physiologic electrical propagation from lead stimulation, right ventricular pacing (RVP) can cause significant ventricular dyssynchrony, and long‐term use of RVP can contribute to pacing‐induced cardiomyopathy (PICM) which is characterized by ventricular systolic dysfunction (Khurshid & Frankel, 2023). Between 6% and 22% of patients who received PPM implantation meet the criteria for PICM within 3–16 years, at a median time of 4.7 years (Kiehl et al., 2016). Among various risk factors, high RVP burden is a well‐acknowledged factor of PICM. The large‐scale studies, including the Mode Selection trial (MOST) and the Dual Chamber and VVI Implantable Defibrillator (DAVID) trial, have demonstrated an association between RVP percentage and worsening heart failure outcomes. A 10% increase in RVP percentage correlated with a 20% rise in HF hospitalization (Sharma et al., 2005), whereas individuals with RVP percentage over 40% exhibited a 2.5‐fold greater risk of HF hospitalization compared to those with RVP percentage below 40% (Sweeney et al., 2003). The precise pathophysiologic processes behind the development of PICM are yet to be fully elucidated.

It has been proposed that mitochondrial function may be a potential underlying mechanism. Mitochondrial dysfunction is associated with a decrease in energy production, reactive oxygen species (ROS) generation, impaired antioxidation, abnormal calcium handling, and the promotion of programmed cell death. These factors all contribute to continuing cellular damage and ultimately resulting in erroneous organ function and myocardial dysfunction (Sabbah, 2020; Zong et al., 2024). Mitochondrial dysfunction has been presented in several cardiovascular diseases, including heart failure and ischemic heart disease (Siasos et al., 2018). Previous animal studies demonstrated that RVP‐induced cardiomyopathy is characterized by diminished mitochondrial quantity, mitochondrial damage, fatty acid accumulation, and lipotoxicity (Lin et al., 2024). Additionally, there is a reduction in the activities of mitochondrial respiratory complexes, particularly complexes III and V, which are essential for ATP production, alongside an increase in apoptosis and a decrease in autophagy and mitophagy (Yu et al., 2019). In contrast, biventricular pacing, a more physiologic pacing compared to RVP, exhibited advantages for mitochondria, along with improvement in cardiac function in animal models (Agnetti et al., 2010; Yu et al., 2019). However, studies involving human subjects in this area have been limited.

The direct evaluation of cardiomyocyte mitochondrial function constitutes the most straightforward method for elucidating the factors that contribute to PICM. Nevertheless, acquiring heart tissue samples is an exceedingly intrusive technique and is impractical for clinical research. Peripheral blood mononuclear cells (PBMCs) offer a less invasive assessment of the impact of RVP, potentially reflecting inflammation and oxidative stress related to the cardiac dysfunction. PBMCs may develop mitochondrial dysfunction while passing through injured cardiac tissue that displays dysfunctional mitochondria (Sauer et al., 2022). Mitochondrial dysfunction in cardiomyocytes can induce cellular stress responses resulting in the recruitment of immune cells and induces mitochondrial dysfunction in PBMCs (Suetomi et al., 2019; West, 2017). Circulating inflammatory cytokines and mitochondrial damage‐associated molecular patterns (mitoDAMPs) generated during pathologic cardiac conditions might directly influence the mitochondrial function of PBMCs (Zhou et al., 2020). Then dysfunctional mitochondria in PBMCs result in heightened inflammation (Tatsumi et al., 2000; Zhou et al., 2011), resulting in endothelial dysfunction, myocardial remodeling, elevated oxidative stress, calcium handling abnormalities, and the promotion of adverse cardiac remodeling (Gulick et al., 1989; Tatsumi et al., 2000). We proposed that evaluating mitochondrial function in PBMCs may serve as a relevant surrogate marker for assessing the impact of RVP, indicating the possible consequences on cardiac and systemic mitochondrial function.

Recognizing that the RVP proportion and exposure duration are significant risk factors for PICM, we aimed to assess the relationship between RVP percentage and duration with various mitochondrial parameters, specifically in PBMCs, to elucidate the adverse effects of RVP on mitochondrial function as a potential underlying mechanism of PICM.

## 2 METHODS

### 2.1 Trial design and setting

This was a cross‐sectional single‐centered study that included patients who underwent PPM implantation with RVP capabilities and followed up at the cardiac implantable electrical devices (CIEDs) clinic at Maharaj Nakorn Chiang Mai Hospital, Thailand.

The study was approved by the ethics committee of the Faculty of Medicine, Chiang Mai University, approval number 058/2566. The investigations were carried out in accordance with the Declaration of Helsinki, including the written informed consent of all participants.

### 2.2 Patients

Inclusion criteria included all of the following: (1) age ≥ 20 years old, (2) presence of a PPM with RVP capability that has been placed for more than 1 month, and (3) left ventricular ejection fraction (LVEF) at enrolment ≥50%.

Exclusion criteria included any of the following: (1) presence of defibrillator lead, biventricular pacing, conductive system pacing, leadless pacemaker, or functional epicardial lead, (2) presence of uncorrected pacemaker malfunction resulting in oversensing or undersensing, (3) end‐of‐life battery status, (4) presence of active CIED infection, or (5) diagnosed with acute coronary syndrome, coronary artery bypass grafting, percutaneous coronary intervention, heart failure hospitalization, or cardioversion within 3 months.

### 2.3 Study protocol and data collection

Patient's baseline characteristics, indication for PPM implantation, and date of implant were collected from electronic medical records. The assessment of LVEF was performed using biplane Simpson's method from echocardiogram done within the past year.

Device interrogation was performed by specialized technicians, nurses, or cardiologists. The percentage of RVP was obtained. The division into two groups based on RVP pacing proportion at a cut point of 40% was prespecified (RVP < 40% and RVP ≥ 40%), as this proportion is indicative of pace dependency and correlates with an increased risk of PICM (Sharma et al., 2005). For the division using the duration after implantation, we used the median duration among our population as the cutoff for the separation.

### 2.4 Outcomes

Twenty mL of blood was drawn in EDTA tubes for a complete blood count (CBC) and metabolic panel, including BUN, creatinine, estimated glomerular filtration rate, electrolytes, and HbA1C. Another 20 mL of blood was collected in EDTA tubes for the assessment of mitochondrial function and oxidative stress in isolated PBMCs. This evaluation encompassed mitochondrial mass, mitochondrial and cellular oxidative stress, basal respiration, maximal respiration, respiratory capacity, proton leak respiration, and ATP production.

### 2.5 Blood measurements

#### 2.5.1 Isolation of blood PBMCs

The isolation of PBMCs was performed by the utilization of Ficoll density gradient centrifugation. The first step of centrifugation included applying a force of 1000 *g* for 10 min. Following this, white and red blood cells were gathered and subsequently reconstituted in a phosphate buffer saline solution (PBS, Merck, Cat# 524650). After this, a mixture of white and red blood cells was placed on Ficoll‐Paque reagent (Sigma, Cat# 10771) and underwent centrifugation at 400 *g* for a duration of 30 min. After the process of centrifugation, the PBMC ring situated near the interface between Ficoll and plasma was collected and subsequently underwent two washes using 10 mL of PBS. The number of PBMCs was quantified using the hemocytometer methodology after the final centrifugation at 1000 *g* for 10 min (Betsou et al., 2019; Brand & Nicholls, 2011).

#### 2.5.2 Mitochondrial mass determination in PBMCs

A total of 2 × 10^5^ PBMCs were stained with MitoSOX™ (Thermo Fisher, Cat# M36008) and co‐incubated with MitoTracker™ Green (Thermo Fisher, Cat# M46750). The cells were incubated at 37°C for 30 min, followed by two washes with PBS. Flow cytometry was then performed to measure fluorescence intensities. MitoSOX™ fluorescence, an indicator of mitochondrial oxidative stress, was detected at an excitation wavelength of 510 nm and an emission wavelength of 580 nm. MitoTracker™ Green fluorescence, an indicator of mitochondrial mass, was detected at an excitation wavelength of 490 nm and an emission wavelength of 516 nm. Fluorescence intensities for both dyes were acquired using a flow cytometer (BD FACSCelesta, BD Biosciences, NJ, USA) and analyzed with an analytical program (BD FACSDiva software, BD Biosciences, NJ, USA).

#### 2.5.3 Cellular oxidative stress determination

A Dichlorohydro‐Fluorescein Diacetate (DCFH‐DA) dye (Sigma, Cat# 35845) was utilized to measure cellular oxidative stress. Stained PBMCs (2 × 10^5^ cells) with 2 μM DCFH‐DA dye at 25°C for 20 min. DCFH‐DA diffused through the cell membrane and was deacetylated by intracellular esterase to a nonfluorescent molecule, which ROS oxidized to dichlorofluorescein. DCF fluorescence intensity was measured at λ ex 485 nm and λ em 530 nm using BD FACS Celesta flow cytometry (San Jose, California, USA) (Tan & Berridge, 2000).

#### 2.5.4 Mitochondrial ROS levels in PBMCs

PBMCs were incubated with 5 μM MitoSOX red staining at 37°C for 30 min. MitoSOX is a fluorogenic dye that selectively detects superoxide in PBMC mitochondria. It emits fluorescence at 580 nm after being stimulated at 510 nm. The fluorescence intensity of MitoSOX‐stained PBMCs was measured using flow cytometry (BD FACS Celesta, CA, USA) to determine mitochondrial ROS levels. Unstained cell controls were incorporated into each sample (De Biasi et al., 2016).

#### 2.5.5 Mitochondrial respiration in PBMCs

PBMCs were utilized to quantify oxygen consumption throughout the implementation of the Mito Stress test via an Agilent Seahorse XFe96 high‐throughput system (Agilent seahorse, Cat# 103015‐100). Base medium was added to PBMCs in a 96‐well plate (Agilent seahorse, Cat# 103792‐100), and baseline respiration was assessed. The ATP production was measured after the complex V inhibitor (oligomycin) was added. Following the addition of the potent oxidative phosphorylation uncoupler (FCCP), the maximum respiration and spare respiratory capacity were ascertained. Finally, the complex I and III inhibitor mixture (Antimycin A and Rotenone) was introduced, followed by the assessment of non‐mitochondrial respiration (Iwata et al., 2022).

### 2.6 Statistical considerations

#### 2.6.1 Statistical analysis

The categorical data were presented as *N* (%). For continuous data, the Shapiro–Wilk test was used to evaluate normality. Mean ± standard deviation (SD) was used to represent continuous variables with a normal distribution. The non‐normally distributed continuous variables were shown as median (interquartile range 25th percentile, 75th percentile). Outcomes were compared between two separate groups using an independent *t*‐test for normally distributed data and the Mann–Whitney *U* test for non‐normally distributed data. To adjust with potential confounding factors, multivariable analyses were conducted using the generalized linear models, which incorporated the prespecified confounding factors that could influence the mitochondrial function, including age, hypertension, diabetes mellitus, chronic kidney disease, coronary artery disease, history of atrial fibrillation, atrial pacing percentage, and baseline characteristics that exhibited differences between groups with *p* <0.1. A 2‐tailed probability level of *p* <0.05 was considered statistically significant. All statistical analyses were performed by IBM SPSS Statistics Version 29.0.1.0 (171).

#### 2.6.2 Sample size calculation

Considering the scarcity of previous data regarding the correlation between mitochondrial function and the percentage or duration of RVP, the objective of this pilot research was to enroll a minimum of 80 patients in our investigation.

## 3 RESULTS

### 3.1 Enrolment

**FIGURE 1:**
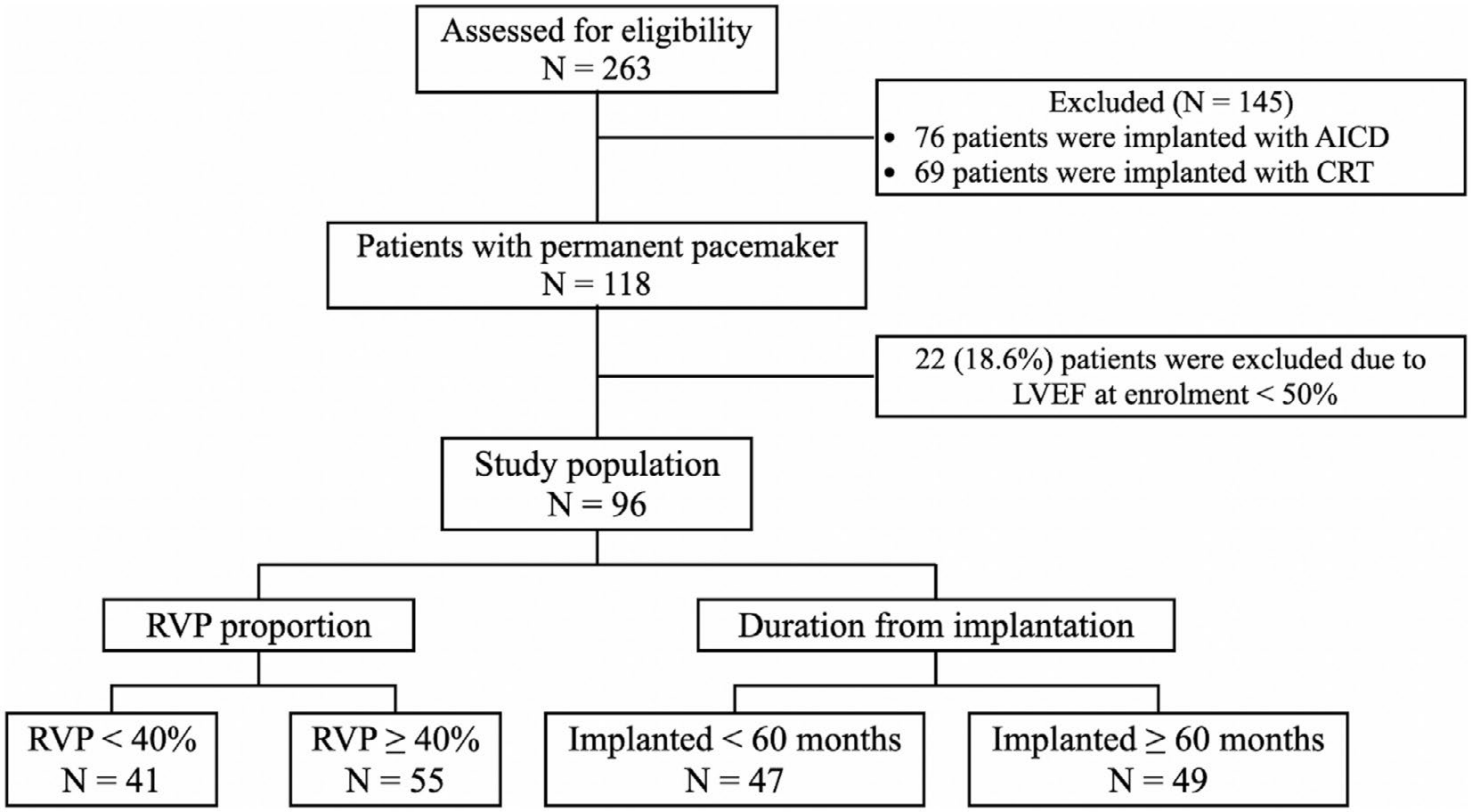
Study flow diagram.

We initially enrolled 263 patients who had implantation of CIEDs and subsequently followed up at our CIED clinic for the main study of mitochondrial function in patients with CIEDs. After excluding individuals according to the exclusion criteria, there were 96 patients entered to the final analysis. The flow diagram of our study is shown in Figure 1.

### 3.2 Baseline characteristics

Tables 1 and 2 present the baseline characteristics of the population and comparison between groups categorized by RVP percentage and duration from implantation, respectively. The mean age of our population was 69 years, with diabetes mellitus being the most prevalent underlying disease. Atrial fibrillation accounted for 18% of the total population. The majority of patients had dual‐chamber pacemakers (89.6%). The predominant indication for pacemaker implantation was atrioventricular block (70.8%). The median RVP percentage of our population was 84%, and more than half of patients were RVP dependent (57.3% had RVP ≥ 40%).

**TABLE 1:** Baseline characteristics comparing between RVP < 40% and RVP ≥ 40% group.

Baseline characteristics	Total (*n* = 96)	RVP < 40% (*n* = 41)	RVP ≥ 40% (*n* = 55)	*p* Value
Age (years)	69 ± 13.4	70.0 ± 12.4	68.3 ± 14.2	0.364
Genders				
Male	45 (46.9%)	11 (26.8%)	34 (61.8%)	<0.001*
Female	51 (53.1%)	30 (73.1%)	21 (38.1%)	
BMI (kg/m^2^)	24.1 ± 4.5	23.8 ± 3.7	24.4 ± 4.9	0.407
Underlying conditions				
DM	63 (65.6%)	27 (65.9%)	36 (65.4%)	0.968
Hypertension	28 (29.1%)	13 (31.7%)	15 (27.2%)	0.621
CKD	9 (9.4%)	2 (4.9%)	7 (12.7%)	0.293
CAD	16 (16.7%)	4 (9.7%)	12 (21.8%)	0.167
AF	18 (18.8%)	9 (21.9%)	9 (16.4%)	0.488
Laboratory results				
Hematocrit (%)	38.9 (35.4, 42.7)	37.6 (35.6, 40.7)	39.2 (34.6, 43.8)	0.208
eGFR (mL/min/BSA1.73m^2^)	70.8 (52.3, 84.9)	74.2 (60.9, 87.2)	65.8 (48.4, 84.9)	0.176
Median time from implantation (months)	64.0 (5.3, 105.8)	64.0 (16.0, 106.5)	58 (1.0. 106.5)	0.445
LVEF at enrollment (%)	66.2 ± 8.2	67.5 ± 7.0	65.2 ± 9.0	0.069
Pacemaker indications				
Sick sinus syndrome	34 (35.4%)	30 (73.2%)	4 (7.3%)	<0.001*
Atrioventricular block	68 (70.8%)	13 (31.7%)	55 (100%)	<0.001*
Second degree	7 (7.3%)	3 (7.3%)	4 (7.3%)	
Advance degree	51 (53.1%)	4 (9.8%)	6 (10.9%)	
Third degree	68 (70.8%)	6 (14.6%)	45 (81.8%)	
Types of pacemaker				
Dual chamber pacemaker	86 (89.6%)	38 (92.7%)	48 (87.2%)	0.509
Single chamber pacemaker	10 (10.4%)	3 (7.3%)	7 (12.8%)	
Modes of pacemaker				
DDD(R)	84 (87.5%)	36 (87.8%)	48 (87.3%)	0.548
VVI(R)	10 (10.4%)	4 (9.8%)	6 (10.9%)	
DDI	1 (1.0%)	0 (0.0%)	1 (1.8%)	
AAI	1 (1.0%)	1 (2.4%)	0 (0.0%)	
Atrial pacing percentage (%)	5 (0.0, 33.0)	24.0 (0.0, 70.4)	3.0 (0.0, 10.0)	<0.001*
Ventricular pacing percentage (%)	84 (2.4, 100.0)	2.0 (0.1, 7.9)	100.0 (97.0, 100.0)	<0.001*

Abbreviations: AF, atrial fibrillation; BSA, body surface area; BMI, body mass index; CAD, coronary artery disease; CKD, chronic kidney disease; DM, diabetes mellitus; eGFR, estimated glomerular filtration rate; kg, kilogram; LVEF, left ventricular ejection fraction; m^2^, square meter; min, minute; mL, milliliter; RVP, right ventricular pacing.

* Significant *p* value (<0.05).

**TABLE 2:** Baseline characteristics and comparison between duration from implantation <60 months and ≥60 months.

Baseline characteristics	Total (*n* = 96)	Implanted <60 months (*n* = 47)	Implanted ≥60 months (*n* = 49)	*p* Value
Age (years)	69 ± 13.4	66.9 ± 12.3	71.0 ± 14.3	0.138
Genders				
Male	45 (46.9%)	22 (46.8%)	23 (46.9%)	0.990
Female	51 (53.1%)	25 (53.2%)	26 (53.1%)	
BMI (kg/m^2^)	24.1 ± 4.5	25.1 ± 5.3	23.3 ± 3.4	0.053
Underlying conditions				
DM	63 (65.6%)	28 (59.6%)	35 (71.4%)	0.222
Hypertension	28 (39.1%)	16 (34.0%)	12 (24.5%)	0.384
CKD	9 (9.4%)	2 (4.2%)	7 (14.3%)	0.160
CAD	16 (16.7%)	6 (12.8%)	10 (20.4%)	0.315
AF	18 (18.8%)	9 (19.1%)	9 (18.4%)	0.922
Laboratory results				
Hematocrit (%)	38.9 (35.4, 42.7)	38.8 (35.5, 41.9)	38.9 (34.1, 43.7)	0.665
eGFR (mL/min/BSA1.73m^2^)	70.8 (52.3, 84.9)	77.0 (52.4, 89.6)	65.8 (49.8, 79.8)	0.136
Median time from implantation (months)	64.0 (5.3, 105.8)	4.0 (1.0, 31.0)	105.0 (89.0. 156.0)	<0.001*
LVEF at enrollment (%)	66.2 ± 8.2	65.5 ± 8.2	66.9 ± 8.3	0.421
Pacemaker indications				
Sick sinus syndrome	34 (35.4%)	16 (34.0%)	18 (36.7%)	0.783
Atrioventricular block	68 (70.8%)	33 (70.2%)	35 (71.4%)	0.896
Second degree	7 (7.3%)	4 (8.5%)	3 (6.1%)	
Advance degree	51 (53.1%)	7 (14.9%)	3 (6.1%)	
Third degree	68 (70.8%)	22 (46.8%)	29 (59.2%)	
Types of pacemaker				
Dual chamber pacemaker	86 (89.6%)	42 (89.4%)	44 (89.7%)	0.944
Single chamber pacemaker	10 (10.4%)	5 (10.6%)	5 (10.2%)	
Modes of pacemaker				
DDD(R)	84 (87.5%)	43 (91.4%)	41 (83.7%)	0.492
VVI(R)	10 (10.4%)	4 (8.6%)	6 (12.3%)	
DDI	1 (1.0%)	0 (0.0%)	1 (2.0%)	
AAI	1 (1.0%)	0 (0.0%)	1 (2.0%)	
Atrial pacing percentage (%)	5 (0.0, 33.0)	3.0 (0.0, 24.0)	5.0 (0.0, 43.0)	0.535
Ventricular pacing percentage (%)	84 (2.4, 100.0)	94.0 (2.5, 100.0)	71.0 (2.5, 100.0)	0.947

Abbreviations: AF, atrial fibrillation; BSA, body surface area; BMI, body mass index; CAD, coronary artery disease; CKD, chronic kidney disease; DM, diabetes mellitus; eGFR, estimated glomerular filtration rate; kg, kilogram; LVEF, left ventricular ejection fraction; m^2^, square meter; min, minute; mL, milliliter.

* Significant *p* value (<0.05).

There was male predominance in the RVP ≥ 40% group, while more females were in the RVP < 40% group. As certain indications are strongly correlated with subsequent RVP percentages, all individuals in the RVP ≥ 40% group had AV block, and the majority of the RVP < 40% group (73.2%) had sick sinus syndrome as their primary pacemaker indication. This also led to a higher atrial pacing proportion among the RVP < 40% group (24.0% vs. 3.0%, *p* <0.001).

The median duration since implantation was 64 months; therefore, we adopted a cutoff of 60 months to categorize the group based on the duration from implantation. There were no significant differences in baseline characteristics between the groups with durations of less than 60 months and those with durations of 60 months or more (Table 2).

### 3.3 RVP percentage and mitochondrial function in isolated PBMCs

**FIGURE 2:**
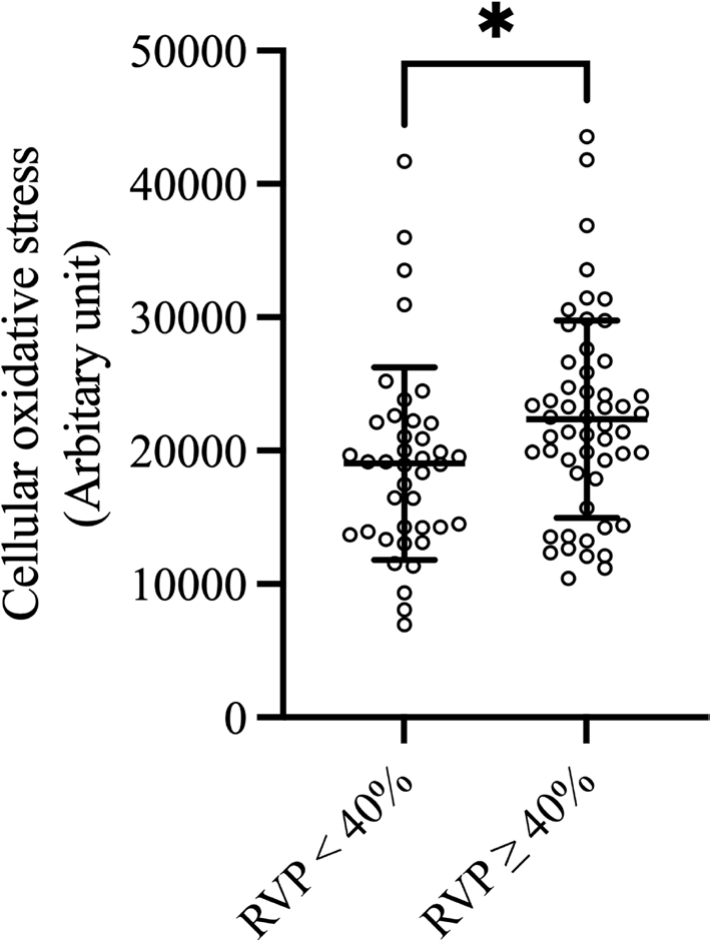
Relationship between RVP proportion and cellular oxidative stress. The scatter plots show individual data points for cellular oxidative stress (Arbitrary unit) in PBMCs from patients grouped by RVP proportion (RVP < 40% and RVP ≥ 40%). Horizontal bars indicate the mean, and error bars represent the standard deviation. *Indicates statistical significance with *p* < 0.05.

Table 3 presents a comparison of mitochondrial function between the RVP < 40% group and the RVP ≥ 40% group. According to our study findings, individuals who had substantial RVP (RVP ≥ 40%) exhibited significantly higher cellular oxidative stress levels compared to those with lower RVP percentages (21,676.0, IQR 17,346.00, 26,067.5 vs. 19,063.0, IQR 14,009.2, 22,110.7 arbitrary unit, *p* = 0.015) (Figure 2).

**TABLE 3:** Comparison of mitochondrial function parameters between the RVP < 40% and the RVP ≥ 40% groups.

Mitochondrial function	RVP < 40% (*n* = 41)	RVP ≥ 40% (*n* = 55)	*p* Value
Spared respiratory capacity (pMol/min)	117.84 (64.44, 220.15)	199.42 (60.39, 215.53)	0.940
Cellular oxidative stress (Arbitrary unit)	19,063.00 (14,009.25, 22,110.75)	21,676.00 (17,346.00, 26,067.50)	0.015*
Mitochondrial oxidative stress (Arbitrary unit)	1513.00 (833.75, 3159.00)	2033.00 (1354.50, 3509.25)	0.234
Mitochondrial mass (Arbitrary unit)	10,883.00 (3299.50, 19,761.00)	16,383.00 (9413.00, 26,433.00)	0.076
Mitochondrial oxidative stress/mass ratio (pMol/min)	0.17 (0.11, 0.28)	0.15 (0.09, 0.23)	0.367
Non‐mitochondrial respiration (pMol/min)	16.34 (11.97, 23.69)	16.32 (14.14, 22.29)	0.706
Basal respiration (pMol/min)	31.53 (18.80, 44.57)	26.90 (18.58, 47.86)	0.999
Maximal respiration (pMol/min)	135.23 (68.75, 238.74)	227.71 (90.99, 236.48)	0.990
Proton leak (pMol/min)	5.18 (2.29, 9.92)	4.96 (2.61, 8.70)	0.980
ATP‐linked respiration (pMol/min)	98.64 (61.91, 103.46)	93.35 (67.52, 102.10)	0.407
Coupling efficiency (pMol/min)	247.49 (105.94, 545.36)	356.18 (94.24, 478.11)	0.970

Abbreviations: ATP, Adenosine triphosphate; min, minute; pMol, picomoles; RVP, right ventricular pacing.

* Significant *p* value (<0.05).

Table 4 presents a multivariable analysis of RVP percentage and cellular oxidative stress. After adjusting for confounding variables, including age, sex, BMI, hypertension, diabetes mellitus, chronic kidney disease, coronary artery disease, history of atrial fibrillation, LVEF at enrolment, atrial pacing percentage, substantial RVP remained an independent risk factor for increased cellular oxidative stress.

**TABLE 4:** Multivariable analysis for the association between RVP percentage and cellular oxidative stress.

Variables	Multivariable analysis		
	Regression coefficient, B (95% confidence interval)	Wald χ^2^ (df = 1)	*p* Value
RVP ≥ 40%	3411.31 (76.93 to 6765.70)	4.02	0.045*
Age	15.61 (−127.02 to 158.25)	0.05	0.830
Male sex	−492.16 (−3690.38 to 2707.06)	0.09	0.763
BMI	−14.09 (−356.32 to 328.13)	0.01	0.936
DM	1773.16 (−2063.43 to 5609.76)	0.82	0.365
Hypertension	3120.46 (−569.26 to 6810.18)	2.75	0.097
CKD	323.79 (−4885.31 to 5532.89)	0.01	0.903
CAD	−1975.65 (−6106.93 to 2155.63)	0.88	0.349
AF	−2218.31 (−6475.21 to 2038.59)	1.04	0.307
LVEF at enrolment	−65.81 (−267.633 to 136.02)	0.41	0.523
Atrial pacing percentage	−8.69 (−57.95 to 40.57)	0.12	0.730

Abbreviations: AF, atrial fibrillation; BMI, body mass index; CAD, coronary artery disease; CKD, chronic kidney disease; DM, diabetes mellitus; LVEF, left ventricular ejection fraction; RVP, right ventricular pacing.

* Significant *p* Value (<0.05).

Incorporating pacemaker indication directly into multivariable analysis would introduce significant multicollinearity, leading to redundancy and potentially unstable model estimates. Pacemaker indication is a strong determinant of RVP, and including both in the same model could obscure the independent effect of RVP on mitochondrial function.

### 3.4 Duration from implantation and mitochondrial function in isolated PBMCs

**FIGURE 3:**
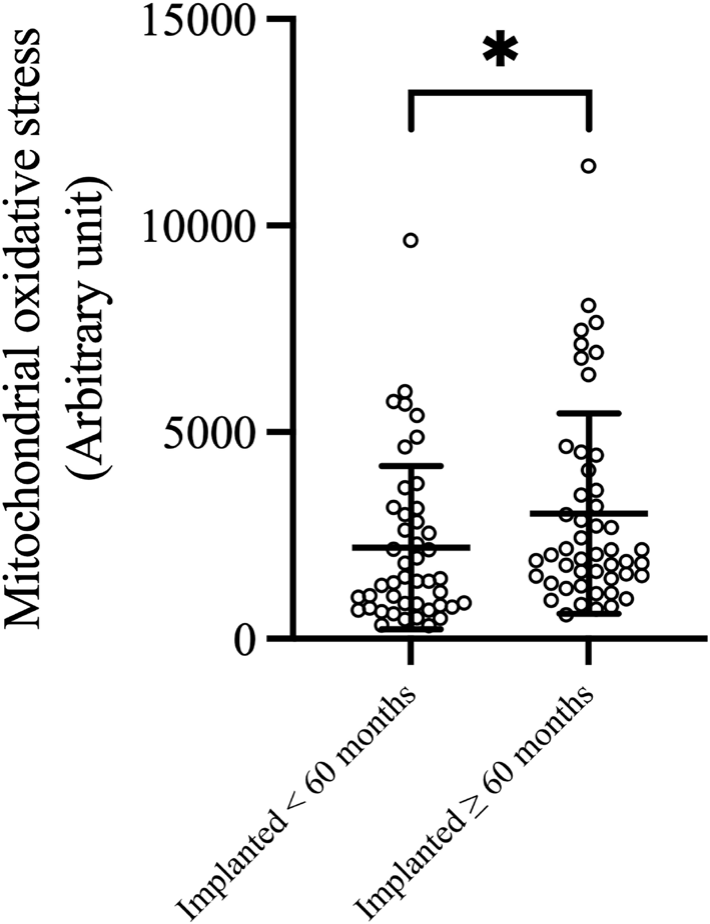
Relationship between duration after implantation and mitochondrial oxidative stress. The scatter plots show individual data points for each mitochondrial oxidative stress (Arbitrary unit) in PBMCs from patients grouped by duration after implantation (duration from implantation <60 months and duration from implantation ≥60 months). Horizontal bars indicate the mean, and error bars represent the standard deviation. *Indicates statistical significance with *p* < 0.05.

Table 5 presents a comparison of mitochondrial function between the duration from implantation <60 months group and ≥60 months groups. The results indicated that patients with a longer duration since implantation or longer RVP exposure exhibited significantly higher mitochondrial oxidative stress compared to those in the shorter duration group (1428.00, IQR 4984.00, 22821.75 vs. 2033.00, IQR 1468.50, 3953.25 arbitrary unit, *p* = 0.030) (Figure 3).

**TABLE 5:** Comparison of mitochondrial function parameters between the duration from implantation <60 months group and ≥60 months group.

Mitochondrial function	Implanted <60 months (*n* = 47)	Implanted ≥60 months (*n* = 49)	*p* Value
Spared respiratory capacity (pMol/min)	109.78 (54.01, 216.17)	204.97 (75.56, 216.45)	0.254
Cellular oxidative stress (Arbitrary unit)	19,358.00 (14,348.75, 23,355.75)	21,664.50 (14,315.75, 24,692.75)	0.223
Mitochondrial oxidative stress (Arbitrary unit)	1428.00 (4984.00, 22,821.75)	2033.00 (1468.50, 3953.25)	0.030*
Mitochondrial mass (Arbitrary unit)	11,192.50 (4984.00, 22,821.75)	17,059.00 (9620.75, 24,850.50)	0.186
Mitochondrial oxidative stress/mass ratio (pMol/min)	0.16 (0.10, 0.22)	0.15 (0.09, 0.28)	0.774
Non‐mitochondrial respiration (pMol/min)	16.43 (12.15, 22.51)	16.27 (12.98, 20.43)	0.861
Basal respiration (pMol/min)	32.62 (18.91, 51.57)	26.20 (17.99, 36.44)	0.214
Maximal respiration (pMol/min)	135.23 (69.27, 235.97)	234.67 (85.81, 239.21)	0.129
Proton leak (pMol/min)	4.76 (2.48, 9.66)	5.11 (2.49, 7.86)	0.890
ATP‐linked respiration (pMol/min)	91.14 (53.43, 102.35)	97.27 (79.01, 103.26)	0.429
Coupling efficiency (pMol/min)	201.21 (88.48, 490.99)	356.18 (117.88, 517.74)	0.254

Abbreviations: ATP, Adenosine triphosphate; min, minute; pMol, picomoles.

* Significant *p* value (<0.05).

The multivariable analysis is shown in Table 6. After adjusting for confounding variables, including age, BMI, hypertension, diabetes mellitus, chronic kidney disease, coronary artery disease, history of atrial fibrillation, and atrial pacing percentage, longer duration since implantation is identified as an independent risk factor for increased mitochondrial oxidative stress.

**TABLE 6:** Multivariable analysis for the association between duration from implantation and mitochondrial oxidative stress.

Variables	Multivariable analysis		
	Regression coefficient, B (95% confidence interval)	Wald *χ* ^2^ (df = 1)	*p* Value
Duration from implantation ≥60 months	957.95 (13.83 to 1902.07)	3.954	0.047*
Age	−12.97 (−29.47 to 55.43)	0.359	0.549
BMI	23.85 (−98.57 to 50.86)	0.392	0.531
DM	−295.22 (−1646.75 to 1056.31)	0.183	0.669
Hypertension	−151.95 (−993.95 to 690.04)	0.125	0.724
CKD	18.53 (−1276.15 to 1313.21)	0.001	0.978
CAD	125.61 (−1318.63 to 1569.85)	0.029	0.865
AF	267.32 (−1434.22 to 1968.85)	0.095	0.758
Atrial pacing percentage	3.41 (−15.88 to 9.07)	0.287	0.592

Abbreviations: AF, atrial fibrillation; BMI, body mass index; CAD, coronary artery disease; CKD, chronic kidney disease; DM, diabetes mellitus.

* Significant *p* value (<0.05).

## 4 DISCUSSION

To the best of our knowledge, our study is among the pioneering studies to evaluate the influence of RVP on mitochondrial function, potentially clarifying the mechanisms behind the detrimental effects of RVP on LV function. Our analysis indicated that among individuals with PPM and normal ventricular function, a substantial RVP frequency, particularly beyond 40% and prolonged RVP exposure beyond 60 months were independently associated with increased cellular and mitochondrial oxidative stress in PBMCs, respectively.

Although we aspire to provide insight into the potential mechanism of PICM, which may arise from RVP impacts on cardiac mitochondria, our study did not directly investigate mitochondria from cardiac tissue. Instead, we determined mitochondrial function in PBMCs, which is less invasive and more practical. While recognizing that PBMCs are distinct from cardiomyocytes in terms of structure and physiological function, several studies have demonstrated a significant relationship between cardiovascular condition and alteration of mitochondrial function within these cells. The investigation conducted in pre‐heart failure patients demonstrated that cardiac disturbances in patients are associated with mitochondrial respiratory dysfunctions of blood mononuclear cells (Li et al., 2015). Another study in patients with heart failure with reduced LVEF demonstrated that patients with more severe heart failure symptoms had significantly higher mitochondrial ROS levels in PBMCs than those with mild symptoms (Shirakawa et al., 2019). Our group recently reported that appropriate atrial pacing setting was associated with improved mitochondrial function in PBMCs and may offer a preventive effect against the onset of atrial fibrillation (Nantsupawat et al., 2024). Mitochondrial dysfunction in PBMCs may be generated by their transit through damaged cardiac tissue with malfunctioned mitochondria. Underlying mitochondrial dysfunction in cardiomyocytes could result in increased cellular stress, including elevated ROS, which subsequently activated the inflammasome/interleukin‐1β (IL‐1β) pathway and facilitated the recruitment of immune cells, thereby inducing mitochondrial dysfunction in PBMCs (Suetomi et al., 2019; West, 2017). The increase in circulating inflammatory cytokines in abnormal cardiac conditions, such as IL‐6, or mitoDAMPs, potentially induced mitochondrial dysfunction in PBMCs. Moreover, dysfunctional mitochondria in PBMCs result in heightened inflammation, including NLRP3 inflammasome activation (Zhou et al., 2011) and IL‐1 generation (Tatsumi et al., 2000). These heightened inflammatory processes lead to endothelial dysfunction, myocardial remodeling, increased oxidative stress, and abnormalities in calcium handling, promoting cardiac remodeling (Gulick et al., 1989; Tatsumi et al., 2000). According to our results, we proposed that elevated oxidative stress in PBMCs among substantial RVP and prolonged RVP exposure groups may reflect the increase oxidative stress in cardiomyocytes or the increased oxidative stress in PBMCs may heighten inflammation and adversely affecting cardiomyocytes, promoting negative remodeling. This could contribute to a fundamental mechanism underlying RVP‐induced cardiomyopathy.

A well‐established significant cause of PICM is RVP‐induced ventricular dyssynchrony, especially with a high percentage and prolonged exposure. Our findings suggested that increased cellular and mitochondrial oxidative stress may serve as an underlying mechanism among this population. Dyssynchrony resulting from RVP leads to variability in loading conditions across different regions (Calle et al., 2021). Clinical data, primarily from cardiac scintigraphy studies, indicated that RVP‐induced dyssynchrony results in increased workload in the lateral wall (Duchenne et al., 2019). This leads to increased uptake of glucose (Duchenne et al., 2019) and fatty acid (Yoshida et al., 1999) in this segment, as well as enhancement in oxidative phosphorylation (Ukkonen et al., 2009). Cardiac oxygen consumption was elevated during periods of RVP‐induced dyssynchrony (Nawa et al., 1992). This may increase the generation of ROS, leading to oxidative stress. Furthermore, the prior transcriptome study examined mRNA profiles from whole blood samples in individuals who underwent PPM implantation and showed that the PRDX1 gene, responsible for encoding the antioxidant enzyme Peroxiredoxin‐1, exhibited downregulation early after pacemaker implantation (Xu et al., 2016). Additionally, the animal study indicated that dyssynchrony from rapid RVP increased the activity of the mitochondrial calcium uniporter (MCU), leading to increased mitochondrial calcium accumulation and reduced mitophagy and autophagy (Yu et al., 2019). Therefore, RVP induces ventricular dyssynchrony, potentially leading to cellular metabolic alteration, incorporated with the depletion of cellular antioxidants, abnormalities in mitochondrial calcium handling, and the removal of dysfunctional mitochondria. This may result in the accumulation of ROS and oxidative stress, potentially impacting PBMCs and cardiomyocytes, and further perpetuated cardiac dysfunction via increasing inflammation through the aforementioned mechanisms.

Currently, there is no evidence supporting the rationale for the differing effects on oxidative stress between substantial RVP and prolonged RVP exposure. The segregation of patients according to RVP percentage and duration may result in the grouping of individuals with varying underlying characteristics, potentially influencing the oxidative stress results. Despite this, we hypothesized that significant RVP could elicit more pronounced acute cellular stress responses. This may indicate heightened cellular oxidative stress, possibly resulting from enhanced activation of inflammatory pathways. Prolonged RVP duration, indicative of prolonged exposure to nonphysiological pacing, may result in persistent and cumulative mitochondrial dysfunction. This may present as elevated oxidative stress in mitochondria.

The principal therapeutic intervention for heart failure resulting from ventricular dyssynchrony, including PICM, is cardiac resynchronization therapy (CRT) (Chung et al., 2023), which may enhance cardiac function by increasing the function of mitochondria. A previous animal investigation was conducted on adult mongrel dogs with LBBB and dyssynchronized heart failure (DHF) to investigate the impact of CRT on mitochondrial function. The study demonstrated that biventricular pacing enhanced ventricular performance in DHF by augmenting mitochondrial energy production and ROS scavenging through elevated Peroxiredoxin‐3 levels (Agnetti et al., 2010). A human metabolomic investigation indicated enhanced mitochondrial performance (elevated Krebs cycle activity and greater metabolic reserve for protein synthesis) and improved antioxidant (increased 2‐hydroxypyridine levels) in the CRT responder group (Nemutlu et al., 2015). These findings corroborate the hypothesis that RVP‐induced cardiomyopathy is associated with mitochondrial dysfunction and increased cellular oxidative stress, which may be ameliorated through CRT.

Our study had some limitations. First, our study was a cross‐sectional analysis of the impact of RVP on mitochondrial function in a group with preserved LV function. It was not aimed at ascertaining whether abnormal mitochondrial function results in a subsequent deterioration of left ventricular function. Our finding that the elevation of oxidative stress with substantial RVP and prolonged RVP utilization may serve as the underlying mechanism for PICM was essentially a hypothesis, and a true causal relationship was not assessed in this investigation. To assess causality, a longitudinal study with baseline measurements of LV function and oxidative stress, followed by repeated assessments over time, would be required. Moreover, there was no comparison between baseline and post‐implant mitochondrial function, leading to the hypothesis that individuals with more severe baseline bradycardia, necessitating greater ventricular pacing, may exhibit increased mitochondrial dysfunction at baseline; thus, no definitive conclusions can be drawn. Secondly, the sample size in our study may be too small to detect the influence of RVP on the other mitochondrial parameters. In the third place, there was lack of a clear definition of impaired versus normal mitochondrial function in PBMCs. We did not have a separate healthy control group to establish a definitive range for normal mitochondrial function. We defined the alteration of mitochondrial function based on statistically significant differences in specific mitochondrial parameters. Lastly, despite our statistical adjustment, complete elimination of the influence of confounding factors may not be possible, and the results may have been affected.

The clinical implication of our study's results is the apprehension over the adverse effects of RVP in patients with PPM, particularly those who are pacemaker‐dependent and necessitate a substantial amount of RVP frequency or duration of RVP. Unnecessary use of RVP should be avoided, and algorithms that encourage the intrinsic ventricular rhythm should be implemented. Physiologic pacing techniques, such as His bundle or left bundle branch pacing (Chung et al., 2023), should be performed instead, as they exhibit clinical benefits compared to traditional pacing. In patients unable to avoid RVP, ventricular function should be assessed annually (Chung et al., 2023), and the evaluation of mitochondrial function in this population may serve as an additional marker to determine who mandates more frequent monitoring or should be considered for alternative physiological pacing implementation to prevent ventricular dysfunction. A therapeutic approach that focuses on lowering the level of oxidative stress may have potential effects in preventing ventricular failure in this population.

Further investigation regarding the impact of RVP on additional domains of mitochondrial function, including mitochondrial dynamics, calcium handling abnormalities, and programmed cell death should be considered. Research on mitochondrial function in novel ventricular pacing techniques, such as His bundle or left bundle branch area pacing, should be conducted to elucidate the mechanisms underlying their advantages.

## 5 CONCLUSION

In individuals who have PPMs and preserved LV systolic function, a significant proportion of RVP and prolonged RVP exposure was independently linked to heightened cellular and mitochondrial oxidative stress, which could contribute to RVP‐induced cardiomyopathy. A larger prospective study may be necessary to validate our findings.

## AUTHOR CONTRIBUTIONS

P.G. collected and rechecked the data, performed statistical analysis, wrote the manuscript, tables, and figures, reviewed and revised the manuscript. T.N., N.P. developed methodology, performed statistical analysis, and reviewed and revised the manuscript. N.A., N.P. collected and rechecked the data prior to the analysis. N.A., S.C.C., N.C., and A.P. revised it critically for important intellectual content. W.W. handled conceptualization, project administration, methodology, reviewed and revised the manuscript, supervision, and funding acquisition. All authors agreed to be accountable for all aspects of the work in ensuring that questions related to the accuracy or integrity of any part of the work are appropriately investigated and resolved. All authors have read and approved the final manuscript.

## FUNDING INFORMATION

This work was supported by the Faculty of Medicine, Chiang Mai University, the Fundamental Fund 2023 project number 4368887, Thailand Science Research and Innovation (TSRI), the Research Chair Grant from the National Research Council of Thailand (NC), the Distinguished Research Professor Grant from the National Research Council of Thailand (SCC), and the Chiang Mai University Center of Excellence Award (NC). The funder had no role in study design, data collection and analysis, the decision to publish, or the preparation of the manuscript.

## CONFLICT OF INTEREST STATEMENT

All the authors declare that they have no competing interests.

## ETHICS STATEMENT

The study was approved by the Ethics Committee of the Faculty of Medicine, Chiang Mai University, approval number 058/2566. The investigations were carried out in accordance with the Declaration of Helsinki, including the written informed consent of all participants.

## Data Availability

The data uploaded in public databases is not included in the informed assent that the participants provided. However, requests for data availability should be sent to bwanwarang@yahoo.com and the Faculty of Medicine, Chiang Mai University Ethics Committee for approval upon request.
